# Factors influencing producers’ implementation of internal teat sealant use on farms with automated milking systems in Ontario—A focus group study

**DOI:** 10.3168/jdsc.2026-1124

**Published:** 2026-09-18

**Authors:** F.J. Guardado Servellon, K.L. Spence, D.L. Renaud, T.J. DeVries, M.W. Brunt, R. Couto Serrenho

**Affiliations:** 1 Department of Population Medicine, University of Guelph, Guelph, ON, Canada, N1G 2W1; 2 Department of Animal Biosciences, University of Guelph, Guelph, ON, Canada, N1G 2W1; 3 MWB Research Consulting Services, Guelph, ON, Canada, N1E 5X7

## Abstract

Automated milking systems (AMS) have been increasingly adopted in the Canadian dairy industry, prompting changes in udder health and dry-off strategies. Use of internal teat sealant (ITS) plays a central role in reducing the risk of IMI during the dry period. Despite this, blanket ITS use remains comparatively lower on AMS farms than in conventional milking systems, suggesting system-specific barriers to its implementation. The objective of this qualitative study was to explore motivations of producers using an AMS for adoption of ITS use as part of their dry-off protocol. Between December 2024 and January 2025, we conducted 4 focus groups with a total of 21 Ontario producers using AMS recruited through veterinary clinics and AMS dealers (median group size: 5 producers, ranging from 4 to 6). The groups intentionally combined producers using (n = 16) and not using ITS (n = 5). The discussions of the focus groups were transcribed verbatim and analyzed using reflexive thematic analysis. Four main themes were generated: producer's assessment of farm suitability; experiences driving ITS use at dry-off; information from trusted professional relationships influencing decision-making; and evaluation of the risks of ITS implementation. Participants' decision-making was strongly influenced by prior experiences by the producer or their peers. Negative experiences included AMS technical issues attributed to ITS residues, and prolonged excretion of ITS in early lactation. In contrast, positive experiences included perceived reductions in mastitis after ITS implementation. Trusted sources, including veterinarians, AMS technicians, and other producers, influenced decision-making, with veterinarians supporting ITS implementation, whereas AMS technicians and other producers could either facilitate or hinder adoption. The risk of a negative outcome (i.e., effects on udder health) after implementation of ITS use was highlighted by several participants. Producers classified as non-ITS users cited the risk of ITS residue buildup and potential AMS malfunction as key barriers for implementation. Conversely, ITS users viewed the practice as essential for reducing mastitis risk. Our results highlight the need for targeted knowledge translation and risk mitigation strategies that specifically address AMS-related concerns and increase the adoption of ITS use in these systems.

## Graphical Abstract

**Figure:**
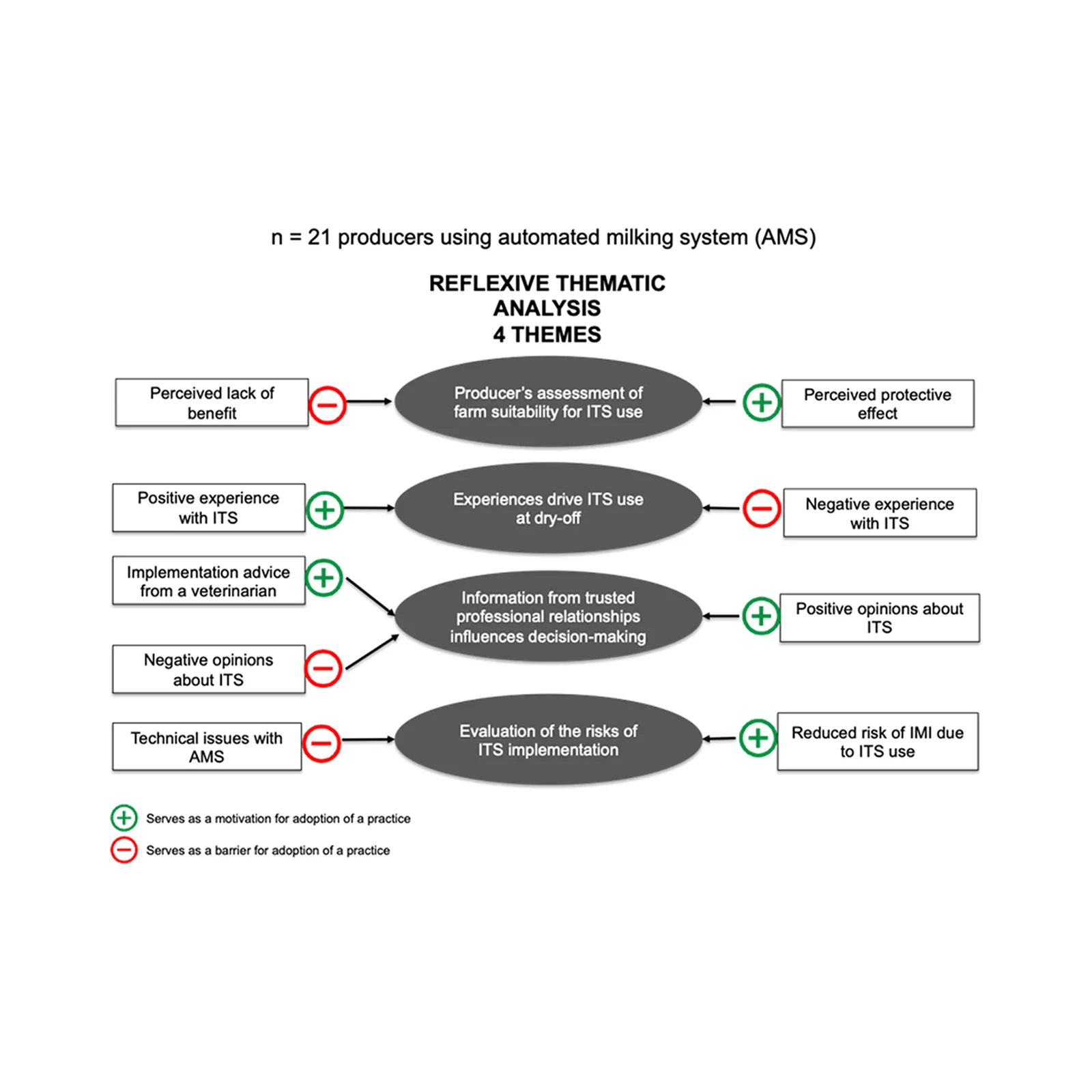

Internal teat sealants (**ITS**) are recognized as an effective strategy that reduces the incidence of IMI during the dry period and early lactation (Rowe et al., 2020). As part of dry cow therapy programs, ITS are recommended as an adjunct to antimicrobial therapy or as a stand-alone option for cows not receiving antimicrobials (Winder et al., 2019). Internal teat sealants act as a physical barrier to compensate for the absence of a keratin seal during the dry period and may have antimicrobial properties (Woolford et al., 1998; Notcovich et al., 2020). Despite demonstrated benefits of ITS use, opportunities remain to further improve its adoption. In a recent Canadian observational study, only 56% of farms (n = 144) across all provinces administered ITS to every cow at dry-off (McCubbin et al., 2023). Among farms that use automated milking systems (**AMS**), blanket ITS use was less likely (odds ratio: 0.19; 95% CI: 0.05–0.73), with only a subset of cows receiving treatment, suggesting that cow-level risk factors and management decisions influence adoption patterns (McCubbin et al., 2023). Internal teat sealant residues might be a factor that influences adoption. Internal teat sealant residues have also been associated with the presence of black spots in aged cheese, which is a concern for the industry (Lay et al., 2007). Additionally, ITS residues have been observed up to 20 DIM (Buckley et al., 2023), which may increase the risk of mechanical or operational issues in AMS and potentially influence producer decision-making within these systems. However, no study has explored the potential factors that might be driving lower use of ITS on AMS farms. Therefore, the objective of this qualitative study was to determine the barriers and motivations that producers using AMS in Ontario, Canada, have for the use of ITS.

This study was approved by the University of Guelph Research Ethics Board (REB-91) and is reported using the Consolidated Criteria for Reporting Qualitative Research (Tong et al., 2007). In qualitative research, our position is not neutral, as our identity and experiences determine how we engage with participants and data (Goundar, 2025). The research team included veterinarians, epidemiologists, animal scientists, and qualitative researchers with experience in dairy health, antimicrobial stewardship, animal welfare, and qualitative methods. We approached our analysis from a critical realist perspective by assuming that an external reality exists independently of our perspectives and by recognizing that our understanding of that reality is shaped by our own experiences and interpretations (Shannon-Baker, 2016).

Participants were recruited using criterion purposive sampling. The criteria for selection were being a dairy producer (owner or manager) over 18 years of age with an AMS farm in Ontario. Producers were identified through veterinarians, researchers, and AMS dealers, who initially approached producers to determine their interest in participating in the study. Contact information was provided to the research team only for producers who expressed interest in participating. One researcher (FGS) contacted producers directly via a phone call, text, or email. Participants' addresses were mapped to identify geographic clusters, and participants were subsequently stratified by location to form groups of 4 to 6. Each group included ITS users and nonusers, to encourage a range of perspectives and stimulate discussion. For this study, ITS users were defined as producers who applied ITS to all cows; none of these participants used ITS selectively. The 4 semi-structured focus groups took place in Southwestern Ontario, hosted in libraries or community centers close to the participants' addresses. Participants were provided with a Can$100 honorarium after the focus group was completed. The sample of 21 participants across 4 focus groups was deemed sufficient to achieve information power, whereby fewer participants are needed when the sample provides substantial information relevant to the study aims (Malterud et al., 2016). This sample size allowed for the development of themes, as the participant group was specific and the research objective was focused on this relatively homogeneous group.

The first focus group was led by author MB, and author FGS led the subsequent 3 focus groups. At the beginning of the focus group, participants were asked closed-ended questions regarding their current dry-off practices, including antimicrobial use at dry-off and experiences with their current protocols. Based on their responses, participants were classified as using selective dry cow therapy (**SDCT**) or blanket dry cow therapy (**BDCT**), and their ITS use status. Participants were then asked about their experiences with ITS as well as current dry-off practices, antimicrobial use at dry-off, challenges related to AMS, and motivators or barriers for using ITS. All authors contributed to the development of the semi-structured discussion guide (available in supplemental materials; see Notes). Focus group discussions were audio recorded using an audio recorder (Sony Corporation, Tokyo, Japan). Verbatim transcriptions were performed by an external company (Otter.ai, December 15, 2024, version). The first author read the transcripts while listening to the recordings 3× to validate accuracy of the data before analysis.

Data were analyzed through reflexive thematic analysis (Braun and Clarke, 2006, 2019). The process is iterative, involving multiple phases such as familiarization with the data, coding, generating themes, and subsequently developing and refining those themes. Throughout this process, meaning is identified and interpreted by using the researcher's subjectivity as an analytical tool. The first author started the analysis by gaining familiarity with the data through listening to the recordings and reading the transcripts. Transcripts were imported to NVivo (version 14.24.2, QSR International Pty Ltd.) and FGS did the initial coding through synthesizing common phrases and key words used by the participants. The first author refined the code names and definitions to better reflect the data, after which patterns across codes were examined to develop the initial themes. At this point, the themes were refined through iterative analysis and discussion with the rest of the research team. The final themes reflect the overarching ideas expressed across the focus groups. Quotes from participants were used to illustrate the themes generated, including number and names (e.g., in square brackets, Theme 1). Producers were identified using focus group number (in square brackets, **FG#**), participant number (**P#**), and use or nonuse of ITS (ITS user or non-ITS user). Any words inserted for clarity are encased in square brackets; ellipses are used where words were removed.

Four focus groups (median group size: 5 producers, ranging from 4 to 6) were conducted in Ontario between December 3, 2024, and January 29, 2025, involving 21 participants from 21 AMS farms. Of the included participants, 16 reported using ITS (BDCT, n = 13; SDCT, n = 2; no antibiotic, n = 1) and 5 did not (BDCT, n = 4; SDCT, n = 1). Participants were kept within their respective focus group (geographic cluster), including at least 1 non-ITS user. Participants included farms using AMS from 3 manufacturers (Lely, n = 10; GEA, n = 7; and Delaval, n = 4). Two groups were manufacturer-specific, and 2 included multiple AMS brands. Focus groups lasted on average 65 min (median 61, range 51–85).

Through reflexive engagement with the focus group transcripts, we developed 4 themes: (1) producer's assessment of farm suitability (2) prior experiences driving ITS use at dry-off, (3) information from trusted professional relationships influencing decision-making, and (4) evaluation of the risks of ITS implementation.

Some participants questioned the perceived benefit of using ITS as part of their dry-off protocol. As one participant explained, “I feel like we're saving time and saving money, and I don't see that it would be a benefit right now to use [ITS] so we kind of take that as a win and direct our money elsewhere” [Theme 1, FG1, P2, non-ITS user]. However, participants also acknowledged that their stance was strongly influenced by their current farm-specific circumstances, as this participant noted: “I've never really considered it, but I think if we were having [udder health] problems, it would be something I would consider” [Theme 1, FG1, P6, non-ITS user]. This highlights that being satisfied with the current udder health status of the herd might be a barrier for implementation of new practices such as ITS use. A producer using ITS highlighted how farm conditions affect his decision-making: “Our cows calve on a compost pack, and then fresh cows are on a compost pack for a week after . . . anytime we are going to do everything we can to give them the best opportunity . . . at least we got some barrier [ITS] of protection” [Theme 1, FG1, P3, ITS user].

Participants frequently described how prior experiences with ITS shaped their current perspectives and management decisions. Several producers raised concerns about the persistence of ITS residues after calving. One participant explained, “You can milk them and three days later, you'll see, still see a little bit of [teat sealant residue] coming out of them” [Theme 2, FG 3, P4, ITS user]. Another farmer described how this persistence affected colostrum management: “There's times where I [forestrip] a fresh cow out a lot. You don't see any more coming, and you get that pail of colostrum out of the side bucket, and it's got [internal teat sealant residues] in it like crazy” [Theme 2, FG 1, P4, ITS user]. Participants further elaborated that these residues created challenges with AMS and overall farm operations. One producer described attempts to mitigate residue issues, stating, “We tried stripping out different [ITS] and a few other things like that. Didn't seem to make a change, and we still had the residue spots in the same old spots on the robot . . . it just wasn't working, so we stopped” [Theme 2, FG 2, P5, non-ITS user]. Another participant highlighted the operational burden associated with managing residues, noting, “We found we were having a high amount of residue in the pipelines and the receiver jars. We were constantly adding the special detergent or shocking the system, and that became pricey, that became another nuisance. So that was also part of the thinking why we just did away with the teat sealant” [Theme 2, FG 4, P4, non-ITS user].

In contrast, some participants described positive prior experiences with ITS, which reinforced continued use. One producer emphasized how positive results have convinced them to continue ITS use: “I don't plan on changing anything. Why change something that's working. I think you look at the cost of [ITS brand name] or whatever it's minimal compared to a mastitis case, especially for me, if I lose the quarter on the cow, right? So, I don't plan on changing it” [Theme 2, FG 2, P4, ITS user]. Others highlighted perceived improvements in protection compared with alternative products used in the past, with one participant noting, “Before we used external teat sealants . . . so compared to that product the internal teat sealant is superior. Especially reading some of the research of how long it takes for a good cow to physically make a barrier in that teat if she's going to make one, like hands down that internal teat sealant can't be beaten” [Theme 2, FG 3, P1, ITS user].

A veterinarian encouraging the use of ITS can play a significant role in shaping producers' decision to use ITS. One participant illustrated this by recalling, “I was influenced by a vet, our herd vet at the time suggested it. We were having a bigger issue. It was a new product, so he recommended it and [we] continue to use it because we felt it was helping with somatic cells and less cases of mastitis at freshening” [Theme 3, FG1, P5, ITS user]. Another noted how positive results can play a role in maintaining a practice after an initial influence: “I probably wasn't there for the discussion, but I'm pretty sure why we use this [ITS] is because the vet told us to and we've been happy with the results” [Theme 3, FG 3, P1, ITS user].

Opinions and experience from technicians and other dairy producers have an influence on participants' perceptions regarding ITS use. Those are particularly influential when a producer is new to AMS: “I know when we first moved into the barn [with an AMS], they [AMS technician] said yeah, I don't know if you want to continue doing that [using ITS]” [Theme 3, FG3, P4, ITS user]. Similarly, another participant noted that ITS use was not a consideration due to the negative opinions: “We were told that teat sealants don't work great in the robots, and then they plug them up. You just have headache after headache of dealing with the sealant in the lines. And so, we didn't even consider doing it when we moved to robots” [Theme 3, FG2, P3, non-ITS user].

One producer voiced the importance of hearing positive experiences from other participants and the potential influence that may have on their opinion: [Talking about what would motivate them to use ITS] “So far, it's working okay for us, but . . . to hear from others today, to hear about where sealants work well maybe bring them back in” [Theme 3, FG2, P5, non-ITS user].

Participants expressed how the risk of having issues with their AMS influenced their decision to not use ITS: “I never even thought about switching to using it [ITS], because I just assumed, like, this is gonna mess with the robot” [Theme 4, FG 1, P6, non-ITS user]. A participant currently using ITS expressed reservation about the potential effects of ITS residues in the AMS: “My experience with it is fine until the day comes where they tell me it's doing something to the inside of my robot . . . I'm not a real fan of it [ITS residues] in pipeline, receiving jars and stuff, because it affects cleaning” [Theme 4, FG1, P4, ITS user].

Participants expressed how reducing the risk of IMI serves as a motivation to use ITS as part of their dry-off protocol. “An ounce of prevention is worth a pound of cure, as the old saying goes. I mean, you do what you can to prevent as much as you can, instead of treatment” [Theme 4, FG 1, P5, ITS user]. Another participant further described this by explaining the financial implications of using ITS: “I think if we just say it's a 10,000-kilo cow, and you take 50 kilos, or even 100 kilos of milk to pay for the teat sealant, I think it's cheap insurance” [Theme 4, FG4, P2, ITS user].

The objective of our study was to explore and describe barriers and motivations that producers using AMS in Ontario have for the use of ITS. Our findings underscore the persistent gap between available research evidence and producers' perceptions relative to ITS effectiveness and safety. Four themes were generated through reflexive thematic analysis, related to farm suitability, prior experiences, input from trusted professional relationships, and perceived implementation risks.

Producers' assessment of their farm's suitability for implementation of ITS use was influenced by multiple factors. Some producers questioned the added benefit to the incorporation of ITS to their dry-off protocol. This perception is consistent with findings from a Swedish web-based questionnaire (n = 340 producers across all milking systems), where the most frequently selected reason for not using ITS (37% of 277 producers using AMS) was the belief that udder health on their farms was already satisfactory (Persson Waller et al., 2021). Such perceptions may reflect a cost-benefit assessment in which the financial investment required to use ITS is viewed as outweighing the anticipated gains. This contrasts with findings from multiple meta-analyses demonstrating an association between ITS use and reduced odds of postcalving IMI, whether used alone or in combination with dry cow therapy (Afifi et al., 2023; Pearce et al., 2023). Together, these findings illustrate a discrepancy between research evidence and on-farm practices, underscoring the importance of exploring the barriers that may exist for implementation.

Prior experiences with specific management practices strongly shaped producers' current perspectives. Multiple participants currently not using ITS reported avoiding their use due to previous challenges, particularly concerns about residue persistence in milking equipment and its perceived contribution to AMS technical issues. In contrast, other producers described positive experiences with ITS and expressed confidence in its continued use. These findings illustrate how experiential knowledge informs perceptions of practice effectiveness and influences implementation (Ritter et al., 2017). A team-based approach including producers and veterinarians in the decision-making process can reduce early implementation issues by providing tools and protocols (Guadagnini et al., 2023). Peer-to-peer learning in which ITS users discuss their experience with other producers can aid successful implementation. This has been suggested by producers in a previous focus group study exploring selective antimicrobial therapy for mastitis and at dry-off (Brunt et al., 2025). These practices can help minimize negative perceptions and experiences to improve long-term adoption.

Trusted professional relationships played an important role in producers' decision-making. Interactions with AMS technicians were influential, with technicians' views on ITS shaping management decisions. This influence may partly explain the lower use of blanket ITS in AMS herds than in non-AMS herds reported in a Canadian survey (McCubbin et al., 2023). This is also relevant to other countries, as a Swedish survey showed that 82% of AMS herds surveyed do not use ITS (Persson Waller et al., 2021). Peer influence was likewise emphasized, a finding consistent with a prior focus group study examining antimicrobial use decision-making among dairy producers (Cobo-Angel et al., 2021). This underscores the importance of providing producers with opportunities to discuss their experiences with one another.

Perceived risk associated with implementing ITS use emerged as a key barrier to adoption. Risk considerations also influenced decisions regarding ITS use. Whereas some participants viewed ITS as a strategy to reduce mastitis risk, others avoided its use due to concerns about potential technical issues with the AMS. Given that ITS residue persistence has been documented in dairy cows despite adherence to best management practices (Buckley et al., 2023), further research is warranted to determine how AMS-specific factors may influence this risk. Knowledge translation efforts that clearly communicate the demonstrated value of ITS use may help increase their adoption. Furthermore, developing and disseminating practical strategies to reduce ITS residues and to optimize dry-off management practices could reduce perceived risk and, in turn, support more confident and appropriate adoption of ITS use.

Due to the purposive sampling method and our sources for potential participants, it is possible that producers with similar views were overrepresented. Moreover, the participants were all from Southwestern Ontario. Although this area represents two-thirds of Ontario dairy producers, our ability to extrapolate to other geographical regions might be limited. Additionally, producers using ITS were more heavily represented in the study than non-ITS users, which may have influenced the range of perspectives captured and the transferability of our findings. This could also have influenced the dynamic of the focus group and reduced the willingness of non-ITS users to voice their opinion. However, the inclusion of multiple AMS brands and both ITS and non-ITS users provided a diverse sample that more closely represents the diverse practices among the source population. Furthermore, the discussion guide included specific questions for non-ITS users, to allow their participation.

Our results indicate that a complex interaction between multiple factors may influence a producer's decision to use ITS as part of their dry cow protocols. Strategies to reduce ITS residues and promote opportunities for producers to share experiences may support broader adoption of ITS use on AMS farms.
